# In-hospital Mortality Characteristics of Women With Acute Myocardial Infarction

**DOI:** 10.4021/jocmr2009.12.1276

**Published:** 2009-12-28

**Authors:** Lea Ann Matura

**Affiliations:** a Bouve College of Health Sciences, Northeastern University, 360 Huntington Ave., Boston, MA, USA 02115. Email: l.matura@neu.edu

## Abstract

**Background:**

Cardiovascular disease continues to be the leading cause of death in women and men in the United States. This study aimed to investigate differences in characteristics between those women who died and survived an acute myocardial infarction (MI).

**Methods:**

This secondary analysis included 109 women. Demographic variables were extracted along with presenting MI symptoms, cardiovascular risk factors (family history of cardiovascular disease, patient history of cardiovascular disease, diabetes, hypercholesterolemia, hypertension, and smoking history), type of MI, time of symptom onset and time of presentation to emergency department (ED) for treatment. Descriptive statistics described the sample, t-tests and chi-square analyzed differences between the groups.

**Results:**

There was a 12% mortality rate for women experiencing an acute MI. The women who died had a mean age of 79 years, approximately 7 years older than those who survived (P = 0.037). The leading MI presenting symptoms were chest pain and shortness of breath. The mean number of cardiovascular risk factors for those who died were 2.15 compared to 2.75 for those who survived (P = 0.063). The majority of those women who survived had a non ST Elevation MI (94%) compared to 54% with a non ST Elevation MI who died. Median time to ED presentation was 242.5 minutes for those who survived compared to 244 minutes who died (P = 0.951).

**Conclusions:**

These data demonstrate a MI mortality profile of women which included an older age, no family history of heart disease reported, and a high rate of hypertension. Those who died reported chest pain and shortness of breath, with several presenting with a syncopal event. In addition, the women represented in this sample had a prolonged presentation time for treatment.

**Keywords:**

Myocardial infarction; Gender; Women; Mortality; Cardiovascular risk factors

## Introduction

Cardiovascular (CV) disease continues to be the leading cause of mortality for women and men in the United States (US), with 4.7 million men and 3.2 million women diagnosed with acute myocardial infarction (MI) in 2006 [1]. A MI occurs where the myocardium has damage due to an inadequate supply of oxygen [2]. Diagnosis is made by the presence of two of the three findings: symptoms characteristic of MI; electrocardiogram (ECG) changes consistent with MI; and/or an increase and decrease of cardiac enzymes. The two major types of MIs are ST elevation MI (STEMI) and Non-ST elevation MI (NSTEMI). STEMIs have the characteristic ST elevation on the ECG indicating myocardial tissue death. NSTEMIs occur when there is temporary blockage of the coronary artery causing myocardial tissue death, but are less extensive than STEMIs.

Since the 1990s, inclusion of women in CV research has determined similarities and differences between the genders. Both genders have the same major risk factors for heart disease, such as cigarette smoking, hypercholesterolemia, hypertension, diabetes mellitus, and family history of CV disease. However, previous research determined of those women suffering a MI only about half had been diagnosed with CV risk factors prior to their MI, demonstrating a lack of screening for risk factors [3]. Studies have shown women and men have different MI symptoms. Women tend to report more back and jaw pain, dyspnea and fatigue in comparison to men who have more chest pain and diaphoresis [4,5].

In addition, previous studies have determined differences in mortality between women and men after an acute MI [6,7]. STEMI mortality continues to be higher than NSTEMI, with higher mortality for STEMI in women and African Americans [8]. Women with NSTEMI who had numerous CV risk factors had a higher mortality rate and received less aggressive treatments than men [9]. Treatment delays in women with an acute MI have been documented [10-13], possibly contributing to morbidity and mortality. An earlier study determined female sex and STEMI as predictors of in-hospital MI mortality [14]. Although the in-hospital mortality rate is 9.4% in the US [15], a recent study determined a higher rate of in-hospital morality among women (12%) compared to men (7%) [16]. In this study we aimed to investigate differences between women who died and those who survived an acute MI.

## Patients and Methods

### Patients

The parent study was a retrospective chart review conducted at a private, 1200-bed academic medical center that include 273 patients, 109 women and 164 men [16]. Inclusion criteria were adult patients with a discharge diagnosis of acute MI. A list of patients with an International Classification of Diseases and Related Health Problems (ICD-9) for acute MI was obtained for the period January 1, 2004 and December 31, 2004. The list contained 348 patients that were randomly selected for inclusion. This secondary analysis included 109 women. The study was conducted after approval from the Institutional Review Board; no identifiers were included or available. Demographic variables were extracted including age, ethnicity, presenting MI symptoms, CV risk factors (family history of cardiovascular disease, patient history of cardiovascular disease, diabetes, hypercholesterolemia, hypertension, and smoking history). A positive smoking history was considered if the patient reported smoking cigarettes within the past year prior to MI admission. STEMIs were further divided into type: anterior/lateral wall MI, anterior wall MI (AWMI), inferior/lateral wall MI, and inferior wall MI (IWMI). Time of symptom onset and time of presentation to the emergency department (ED) were extracted and recorded from the ED intake form. All data were extracted by the principal investigator (LAM).

### Statistics

Data were analyzed using SPSS 17. Descriptive statistics including means, medians and standard deviations were used to describe the sample. The t test analyzed differences in continuous variables. Chi-square analysis was used to determine differences in categorical variables. An alpha of 0.05 was set.

## Results

### Demographics

For the overall sample, the mean age of women was 73 years (SD = 14.54) (Table 1). Sixty four percent were Caucasian (n = 70). The leading presenting symptoms for the group were chest pain (n = 54, 50%) and shortness of breath (n = 23, 21%). A majority of the women reported a prior history of cardiovascular disease (n = 93, 85%). Over half of the patients had hypercholesterolemia with 91% reporting a history of hypertension. Twelve percent of the women died during the MI hospitalization (n = 12). The mean age for those who survived was 72 years (SD = 14.86) compared to those who died with a mean age of 79 (SD = 10.27), P = 0.04. The majority of those who died were Caucasian (62%).

**Table 1:** Demographic and Cardiovascular Risk Factors

	Total Females		Females Survived		Females Died		P Value
	(n = 109)		(n = 96)		(n = 13)		
Age	Mean = 73		Mean = 72		Mean = 79		0.037
	(SD = 14.54)		(SD = 14.86)		(SD = 10.27)		
Ethnicity	n	%	n	%	n	%	< 0.0001
Caucasian	70	64	62	66	8	62	
African American	25	23	23	23	2	15	
Hispanic	6	6	4	4	2	15	
Asian	1	1	0	0	1	8	
Other	7	6	7	7	0	0	
Leading Presenting Symptoms	n	%	n	%	n	%	0.021	
Chest pain	54	50	51	53	3	23	
Shortness of breath	23	21	21	22	2	15	
Syncope	5	5	1	1	4	31	
Risk Factors	n	%	n	%	n	%	< 0.0001
Family history heart disease	15	14	15	15	0	0	
Patient history heart disease	93	85	82	85	11	85	
Diabetes	30	28	28	29	2	15	
Hypercholesterolemia	51	47	47	49	4	31	
Hypertension	99	91	88	92	11	85	
Smoking history	4	4	4	4	0	0	
Mortality	13	12					

*t*-test analyzed differences between continuous variables; chi-square analyzed differences between categorical variables.

### Cardiovascular risk factors, time to ED presentation and mortality

Six major CV risk factors were recorded. Of those women who survived a majority had a history of heart disease (85%) and hypertension (92%), with about half reporting hypercholesterolemia (49%) (Table 1). The only women who reported smoking were those who survived the acute MI. In contrast, those women who died had no report of a family history of heart disease. Eighty five percent of those who died had a history of heart disease and hypertension. The mean number of CV risk factors for those who died were 2.15 compared to 2.75 for those who survived (P = 0.063).

Of the 109 women included in the study, 10 had their MIs after admission to the hospital. Therefore, they could not be included in the time difference between symptom onset and presentation time to the ED. The median time of onset for the 99 women was 244 minutes. Of the 88 who survived the median time to ED presentation was 242.5 minutes compared to 244 minutes for the 11 who died. Comparison of mean ED presentation time differences was not significantly different (P = 0.951).

### Type of MI, presenting MI symptoms and mortality

The majority of those women who survived had a NSTEMI (94%) compared to 54% with a NSTEMI who died (Table 2). Of those who died, 3 had an AWMI and 3 had IWMI. In comparison, in those who survived one had an anterior/lateral wall MI, 5 AWMI, and one inferior/lateral MI. Over half (53%) of those women who survived had chest pain as their presenting MI symptom and 22% had shortness of breath. For those who died 23% had chest pain; 15% reported shortness of breath; and 31% had syncope.

**Table 2:** MI Type and Mortality

	Females Survived		Females Died		P Value
	(n = 96)		(n = 13)		
MI Type	n	%	n	%	< 0.0001
STEMI	90	94	6	46	
NSTEMI	6	6	7	54	

MI: myocardial infarction; STEMI: ST elevation MI; NSTEMI: Non-ST elevation MI.

## Discussion

The results of this study described similarities and differences between those women who survived an acute MI and those who died. Those women who died were about 7 years older than those who survived, yet the mortality group exceeded US female life expectancy rates. When these data were collected the mean age of those who died was 79 years; the average life expectancy age for women in the US was 77.8 years [17]. The majority of women in this sample were Caucasian, including those who died. Only 15% of those who died were African American. Therefore, no sub analysis could be performed to determine if mortality was higher in African Americans as previously reported [8].

More CV risk factors increase the likelihood of suffering a MI, especially in women [18]. Interestingly, those who died actually had lower mean CV risk factors. Our study showed that the majority of those who died had hypertension and a history of heart disease. For history of heart disease, there was not a breakdown into subcategories to determine what diseases constituted heart disease history, such as previous MI or acute coronary syndromes. Although a breakdown was not collected on subgroups for those who reported heart disease, prior heart disease could have contributed to death. The diagnosis of hypertension was considerably high, 85%. In the US, the total female population has a 32.1% hypertension rate [15]. One interesting finding was none of the women who died reported a family history of heart disease. Knowing the genetic and familial contribution to heart disease this is surprising. Some explanations may be related to participants not knowing their specific family history. Although it was a very small percentage, of those who reported smoking in the past year, none of these women were in the group that died.

The women who died had different presenting symptoms than those who survived. About one-third of those who died presented with a syncopal episode, compared to only one (1%) participant in the group who survived. A previous study determined that using a rule out MI protocol for those who reported syncope as a presenting symptom to the ED yielded a low diagnosis of MI [19]. Although there are multiple etiologies of syncope, MI is a possible cause and in our study an initial symptom of syncope resulted in a MI diagnosis and ultimately these women died. Although previous studies have determined that women experience different MI symptoms than men [5], our study demonstrated that over half of those who survived had chest pain and one-fourth with chest pain died. The presentation of chest pain may have prompted expedient MI care than other less classic MI symptoms.

Time from MI symptom onset to presentation for treatment is a critical time period. The Golden Hour is considered the first 60 minutes after MI onset where reperfusion of the myocardium can prevent mortality, an ultimate 120 minute goal for reperfusion with either fibrinolysis or percutaneous coronary intervention (PCI) is recommended [20]. Door to balloon refers to the time a patient with a STEMI enters the ED to time to first PCI balloon inflation which is recommended in 90 minutes or less. Door to needle refers to the time the patient arrives to the ED with a STMEI to time fibrinolysis infusion begins, with a goal of one hour or less [20]. Fibrinolysis is recommended to begin within 180 minutes of MI. Both the mortality (P = 0.013) and survival (P < 0.0001) groups significantly fell out of the 120 minute goal to reperfusion. The mean 330 minutes in the group that died may have ultimately contributed to their death. Continual education of MI symptom identification and prompt medical treatment is necessary to improve the time to treatment intervals.

Although STEMIs generally have a higher mortality than NSTEMI [8], there was an approximate 50/50 split in STEMI versus NSTEMI mortality. As previously stated, reperfusion is ideally initiated in the first 120 minutes of an acute MI [20]. With the increased presentation time to the ED, the 120 minute treatment timeframe could not be achieved in this sample. Treatments and time to treatment (door to balloon or door to needle) were not collected in the original study.

In summary, limitations to this study include the retrospective design and the small number of women in the mortality group along with the limited ethnic diversity. We did not collect or control for treatments which could contribute to mortality. These data demonstrate a MI mortality profile of women which included an older age, no family history of heart disease, and a high rate of hypertension. Those who died did have chest pain and shortness of breath, with several presenting with a syncopal event. In addition, the women represented in this sample had a prolonged presentation time for treatment, which may have contributed to mortality. More research, including treatments, needs to be conducted to continue to improve mortality rates among women with MI.
